# Supplementary material for: A well-timed shift from local to global agreements accelerates climate change mitigation
Source: Nat Commun. 2021 May 18;12:2908. doi: 10.1038/s41467-021-23056-5 (PMC8131631; doi:10.1038/s41467-021-23056-5)
Supplement: Supplementary file 1 — Supplementary Information [file 41467_2021_23056_MOESM1_ESM.pdf]

Supplementary Information for: A well-timed shift from local to global agreements accelerates climate change mitigation

Vadim A. Karatayev, Vítor V. Vasconcelos, Anne-Sophie Lafuite, Simon A. Levin, Chris T. Bauch, Madhur Anand

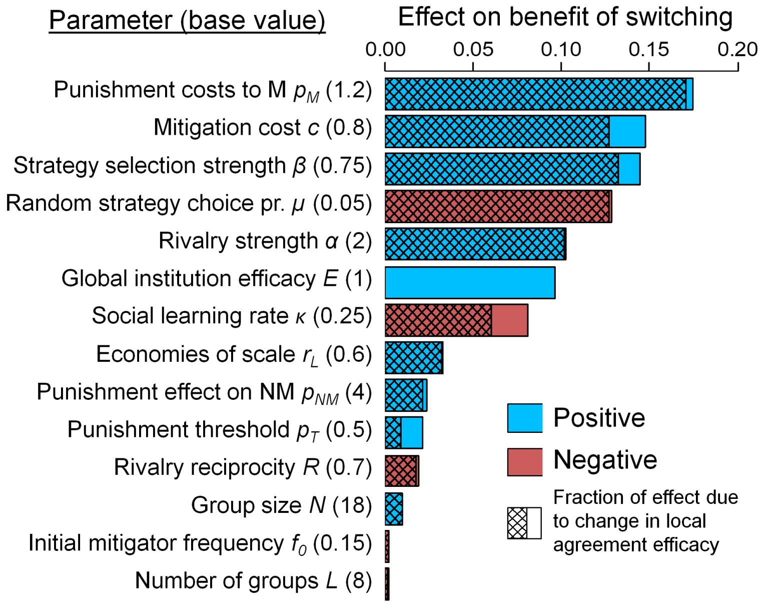

Supplementary Figure 1. Drivers of the benefits of shifting from local to global agreements over local agreements only. Blue (red) bars denote greater (lower) benefits of shifting with an increase in each parameter from 80% to 120% of its base value. Hashes on each bar denote the proportion of each change in shifting benefit caused by a change in mitigation adoption rate using local agreements only, and non-hashed areas denote the proportion of each change in shifting benefit caused by a change in mitigation adoption rate after global agreements begin. We quantify shifting benefits as the proportional increase in total mitigation over the first 625 time steps over 120 replicates.

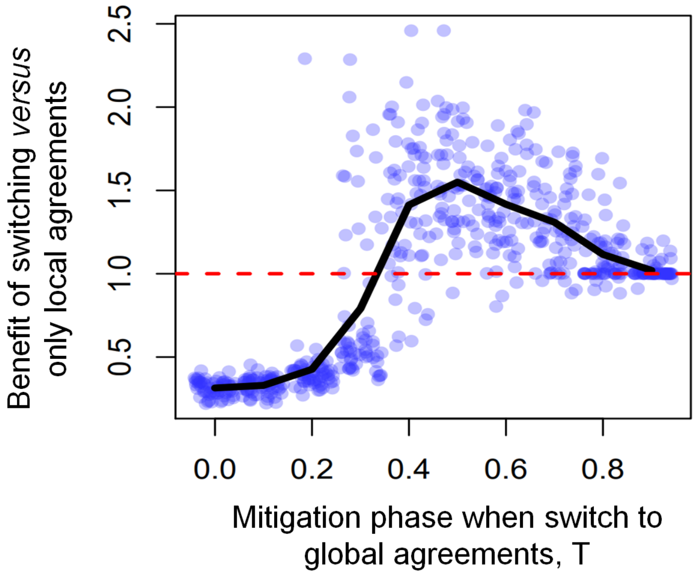

Supplementary Figure 2. Shifting from local to global agreements as soon as global agreements can be established provides the greatest mitigation benefits. Total mitigation achieved by time step 625 when shifting from local to global agreements relative to mitigation when using local agreements only over a range of shifting thresholds T (i.e., a net benefit of shifting for values > 1). Low (high) T values represent a shift to global agreement attempts after few (most) players world-wide have adopted a mitigation strategy. In all cases, the quorum of mitigators required to enact local and global agreements is $p_T = 0.5$, points denote results for individual replicates, lines denote means across replicates, and parameters correspond to the model with economies of scale and rivalry.
